# Midbrain-Hindbrain Boundary Morphogenesis: At the Intersection of Wnt and Fgf Signaling

**DOI:** 10.3389/fnana.2017.00064

**Published:** 2017-08-03

**Authors:** Holly C. Gibbs, Ana Chang-Gonzalez, Wonmuk Hwang, Alvin T. Yeh, Arne C. Lekven

**Affiliations:** ^1^Department of Biomedical Engineering, Texas A&M University College Station, TX, United States; ^2^Department of Materials Science and Engineering, Texas A&M University College Station, TX, United States; ^3^School of Computational Sciences, Korea Institute for Advanced Study Seoul, South Korea; ^4^Department of Biology, Texas A&M University College Station, TX, United States

**Keywords:** MHB, mes/r1, Wnt, Fgf, constriction morphogenesis, two-photon fluorescence, image analysis, zebrafish

## Abstract

A constriction in the neural tube at the junction of the midbrain and hindbrain is a conserved feature of vertebrate embryos. The constriction is a defining feature of the midbrain-hindbrain boundary (MHB), a signaling center that patterns the adjacent midbrain and rostral hindbrain and forms at the junction of two gene expression domains in the early neural plate: an anterior *otx2/wnt1* positive domain and a posterior *gbx/fgf8* positive domain. *otx2* and *gbx* genes encode mutually repressive transcription factors that create a lineage restriction boundary at their expression interface. Wnt and Fgf genes form a mutually dependent feedback system that maintains their expression domains on the *otx2* or *gbx* side of the boundary, respectively. Constriction morphogenesis occurs after these conserved gene expression domains are established and while their mutual interactions maintain their expression pattern; consequently, mutant studies in zebrafish have led to the suggestion that constriction morphogenesis should be considered a unique phase of MHB development. We analyzed MHB morphogenesis in *fgf8* loss of function zebrafish embryos using a reporter driven by the conserved *wnt1* enhancer to visualize anterior boundary cells. We found that *fgf8* loss of function results in a re-activation of *wnt1* reporter expression posterior to the boundary simultaneous with an inactivation of the *wnt1* reporter in the anterior boundary cells, and that these events correlate with relaxation of the boundary constriction. In consideration of other results that correlate the boundary constriction with Wnt and Fgf expression, we propose that the maintenance of an active Wnt-Fgf feedback loop is a key factor in driving the morphogenesis of the MHB constriction.

## Introduction

The midbrain-hindbrain boundary (MHB), also called the isthmic organizer, has piqued the interest of developmental biologists for decades. Characterized by a conspicuous constriction in the developing neural tube, the MHB, located at the interface of the midbrain and hindbrain neuromeres, is well known to function as a signaling center responsible for patterning cell fates anteriorly in the midbrain and posteriorly in the cerebellum (Wurst and Bally-Cuif, 2001; Raible and Brand, 2004; Dworkin and Jane, 2013). The constriction is particularly evident in the dorsal neural tube and defines the posterior midbrain tectum and the hindbrain cerebellum. The MHB constriction also separates ventricular regions within the neural tube lumen, with the midbrain ventricle anterior to the constriction and the hindbrain ventricle behind. The MHB thus represents a crucial dividing point in the developing brain with characteristic morphological features which are critical for several MHB functions: as a signaling center, as a guide for neuronal migration and axon pathfinding (Volkmann et al., 2010), and as a physical separation of brain ventricles (Lowery et al., 2009). What is less well understood is the link between the mechanisms responsible for MHB specification and patterning, and between the signaling molecules that provide its signaling center activity and the constriction morphology that invariantly accompanies vertebrate MHB development. In other words, why is there always a neural tube constriction at the MHB, and is this morphology a cause, or consequence, of MHB function?

The mechanisms behind MHB specification and function are of interest on multiple levels. First, model organism studies have shown that defects in specification and patterning of the MHB lead to major deficiencies in the brain, such as the absence of midbrain, loss of cerebellum, and overgrowth of the midbrain tectum (McMahon and Bradley, 1990; Thomas and Capecchi, 1990; Buckles et al., 2004). Second, advances in magnetic resonance imaging (MRI) have enabled new analyses of human midbrain-hindbrain malformations (Doherty et al., 2013). These new imaging studies are revealing a surprising number of human central nervous system deficits that likely are caused by aberrant developmental patterning, such as the association of septo-optic dysplasia with chromosome 14 deletions, which include the neural patterning gene, *otx2* (Severino et al., 2014). Identifying potential causes of these severe nervous system diseases requires a thorough understanding of the developmental mechanisms behind midbrain-hindbrain development.

As demonstrated by mouse mutants and zebrafish reporter lines, the MHB is specifically positioned within a domain of the early neural plate referred to as mes/r1 in mouse (Zervas et al., 2004) or the midbrain hindbrain domain (MH) in zebrafish (Tallafuss and Bally-Cuif, 2003). These studies show that the early mesencephalon (mes) and rhombomere 1 (r1) in the anterior hindbrain are genetically co-specified, and the MHB defines a balance point between these midbrain and hindbrain divisions. Besides positioning the future MHB, the balance point also represents an interface between Wnt ligand expressing progenitors of the posterior mesencephalon and Fgf ligand expressing progenitors of the anterior rhombencephalon, which interact in multiple ways throughout the specification and morphogenesis of mes/r1 and the MHB. Thus, an important question that is not yet sufficiently answered is what is the significance of the Wnt-Fgf interface at the MHB to mes/r1 development?

## Midbrain Hindbrain Domain Morphogenesis and Patterning

**Figure 1:**
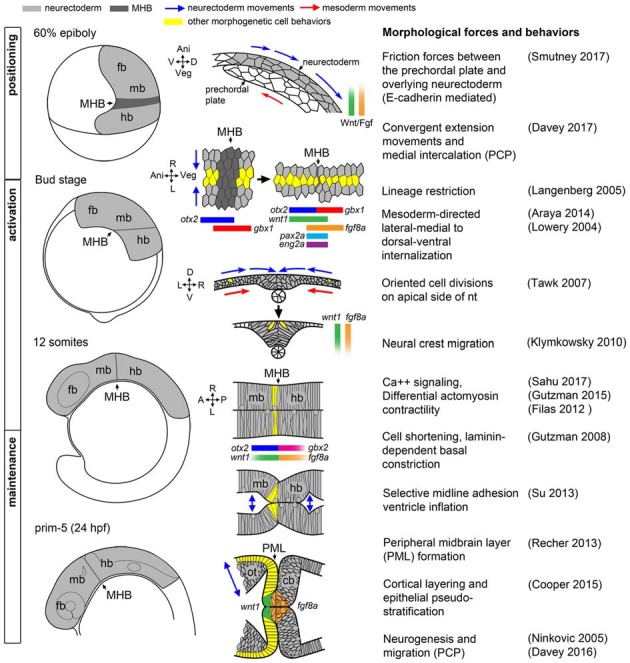
Morphogenetic and molecular ontogeny of the midbrain hindbrain boundary (MHB) in zebrafish embryos. Left column: schematic diagrams of zebrafish embryos, lateral view, at stages indicated on left. Gray shading highlights the brain primordium. Middle column: diagrams of pertinent morphogenetic movements. Orientation is indicated on the left side of each diagram. Examples of each morphogenetic property can be found in the corresponding references in the right column. fb, forebrain; mb, midbrain; hb, hindbrain.

To appreciate the difficulty of dissecting the role of Wnt and Fgf signaling families in the morphogenesis and patterning of the MH by the isthmic organizer, and to begin to identify processes whose disruption would result in neurological disorder, it is helpful to first have a clear picture of how the MH takes shape. A model of the current morphological and molecular ontogeny of the MH region in zebrafish is shown in Figure 1.

### Positioning

The first critical step in MH morphogenesis is correct positioning of the neural primordium on the body axes such that spatiotemporal positioning cues can properly “posteriorize” the nervous system, that is, establish anteroposterior positional information after neural induction. Neural primordium positioning is mediated in part by the mechanical coupling of the presumptive neurectoderm to the involuted, anteriorly-migrating prechordal plate of the axial mesoderm via friction forces generated by the cell adhesion molecule E-cadherin, which is coupled on its cytoplasmic end to the canonical Wnt effector molecule, β-catenin (Smutny et al., 2017). In this study, uncoupled ectodermal cells anterior to the presumptive neurectoderm “flowed” laterally, posteriorly, and then medially. These complex “vortex” migrations of presumptive neurectodermal cells are presumably part of convergent extension movements that rely on non-canonical Wnt/PCP signaling to facilitate medial intercalation and anterior-posterior axis lengthening (Heisenberg et al., 2000; Davey and Moens, 2017). What is remarkable is that on their tumultuous journey, presumptive neurectodermal cells are precisely exposed to a complex molecular program that includes posteriorizing Wnt and Fgf signals emanating from the blastoderm margin (reviewed in Green et al., 2015; Tuazon and Mullins, 2015), resulting in a correctly patterned neural plate. One particularly remarkable and unknown aspect of this process is how early cell responses to Wnt and Fgf ligands occur during the complex morphogenetic changes of gastrulation.

Several lines of evidence suggest that Wnt and Fgf ligands expressed in the nascent paraxial mesoderm function as morphogens by establishing concentration or activity gradients that generate anteroposterior positional information that is interpreted into patterned cell fates (e.g., Cox and Hemmati-Brivanlou, 1995; McGrew et al., 1995). A crucial function in particular for Wnt ligands in polarizing the neural plate has been established from experiments in zebrafish, Xenopus, and chick (Kiecker and Niehrs, 2001; Nordström et al., 2002; Dorsky et al., 2003; Rhinn et al., 2005). The mechanism by which graded Wnt activity is established is not yet known, though recent results suggest delivery of Wnt ligand via filopodia from paraxial mesoderm progenitors may be a major mode of transport (Stanganello et al., 2015). This differs from Fgf in this context, which has been shown to freely diffuse from its paraxial mesoderm source and form a gradient through a source-sink mechanism (Scholpp and Brand, 2004; Yu et al., 2009).

Of importance to the position of the MHB organizer, specifically, is the activation of the transcription factor *gbx1* in the posterior neural plate by Wnt8a (Rhinn et al., 2005, 2009), which antagonizes independently activated *otx2* expressed in the forebrain and midbrain (Kurokawa et al., 2012). In the zebrafish, these domains overlap slightly at 60% epiboly but subsequently become mutually exclusive by 80% epiboly (Rhinn et al., 2003), while in mice there is initially a gap between Otx2 and Gbx2 that closes (Wurst and Bally-Cuif, 2001). In either case, there is a territory of cells that appears to be uncommitted to either the midbrain or hindbrain compartment that is consistent with observed variability in fate mapping experiments conducted before and after gastrulation (Woo and Fraser, 1995). However, strong evidence exists showing that by the time segmentation is underway in zebrafish (Langenberg and Brand, 2005), chick (Tossell et al., 2011a,b), and mice (Sunmonu et al., 2011), the MHB is lineage restricted and cells in the midbrain and hindbrain compartments do not intermix, though this conclusion has not been without controversy (e.g., Jungbluth et al., 2001). Though proper compartmentalization is important for the later establishment of appropriately sized neural progenitor pools in the MH, it remains an open question whether a physical or molecular mechanism precipitates lineage restriction at the MHB. A report from chick suggests there is a posterior shift in the position of the Otx/Gbx interface, such that it only coincides with the physical MHB constriction at later stages in development (Hidalgo-Sánchez et al., 2005). In zebrafish and in mice, however, the consensus is that the initial Otx/Gbx boundary definitively marks the future MHB constriction prior to when it becomes morphologically visible. Notch signaling, which classically causes cells to make such boundary decisions by amplification of small stochastic differences in gene expression followed by cell sorting, has been implicated in sorting Otx and Gbx cells at the MHB in chick (Tossell et al., 2011b). Reports from mice and medaka suggest other genetic interactions may refine the boundary, as Gbx2 interacts with Groucho repressors and can directly compete with POU transcriptional activators of Otx2 (Heimbucher et al., 2007; Inoue et al., 2012). Intercellular actinomyosin networks that have been shown to drive morphogenesis, such as during mesoderm invagination in *Drosophila*, may also play a physical role in lineage restriction at the MH (Kasza and Zallen, 2011) or possibly regional changes in cortical actin tension cause cells to sort to one side of the boundary or another (Heisenberg and Bellaïche, 2013).

### Activation

Shortly after the positioning phase during gastrulation, a suite of MHB genes are activated in distinct domains around the *otx2/gbx* boundary as the neural plate undergoes neurulation to form the neural tube. Expression of *wnt1* anteriorly and *fgf8a* posteriorly to the presumptive MHB (that is still not morphologically obvious) reinforce the *otx2/gbx* interface while *her5*, *eng2a*, and *pax2a* are expressed on both sides of the boundary (Rhinn and Brand, 2001; Buckles et al., 2004). Which molecules and/or forces activate these core members of the more extensive MHB genetic program remains poorly characterized, and, surprisingly, this activation program can occur in the absence of at least parts of the positioning machinery (Su et al., 2014). Once activated, the specific roles of each gene in promoting subsequent development within the MH (beyond providing spatial cues) is also not well understood, though several components of the MHB program appear to have roles in both fate specification and morphogenesis within the MH (Dworkin and Jane, 2013). For instance, *her5* is known to inhibit neurogenesis during segmentation (Tallafuss and Bally-Cuif, 2003; Ninkovic et al., 2005) and to subsequently promote neural stem cell identity in adult zebrafish (Chapouton et al., 2006).

The establishment of the Wnt/Fgf signaling interface, however, is certainly crucial to the development of the MH. Both Wnt1^−/−^ and Fgf8^−/−^ mice fail to develop the entire MH region (McMahon and Bradley, 1990; Chi et al., 2003). In zebrafish, loss of several redundant Wnts (*wnt3*, *wnt3a*, *wnt1* and *wnt10b*) recapitulates a similar phenotype (Lekven et al., 2003; Buckles et al., 2004) and the zebrafish *fgf8a* mutant *ace* lacks a cerebellum and MHB constriction, though the midbrain is present but unpolarized, resulting in aberrant retinotectal projections (Picker et al., 1999). Fgf8 has been deemed the most important “organizing molecule” based on results from implanting Fgf8-soaked beads at sites anterior and posterior to the MHB. In these experiments, Fgf8 was sufficient to induce tectal and cerebellar structures and an underlying Otx/Gbx boundary, while similar experiments for Wnt1 showed no significant re-patterning of the surrounding tissues (Martinez et al., 1999). Indeed, no gain of function analysis for all the other major MHB molecules in any organism has yielded such striking results. However, a study in which Otx2 and Fgf8 were simultaneously knocked down has challenged the idea that Fgf8 is required to pattern cell fates in the MHB. Foucher et al. (2006) showed that in the absence of Fgf8, if Otx2 levels were depleted, cerebellar neurons were able to successfully differentiate, though MHB morphology was abnormal in these embryos. Recent analysis of *otx;gbx;fgf* embryos also suggests that robust cerebellar differentiation requires Fgf (Su et al., 2014).

During MHB program activation, the process of primary neurulation, in which the neural plate coalesces on the dorsal midline, is ongoing (Lowery and Sive, 2004). During this process, the medial-lateral organization of the neural plate is transformed to a ventral-dorsal orientation (Schmitz et al., 1993). It is worth mentioning that although the subsequently developing MHB constriction has been studied primarily in reference to the A/P axis, it is not uniform on the D/V axis of the neural tube, which may reflect graded or inhomogeneous Wnt/Fgf activity along the D/V axis and integration with dorsoventral patterning signaling activities (Lekven et al., 2003; Puelles et al., 2003). Thus, the MHB literature is largely focused on organizer activity in the alar region of the MH with relatively little known of the basal tegmentum.

Shortly after the neural tube is formed and the neural crest begins to migrate, the MHB constriction becomes a visible morphological feature as brain ventricles begin to form. In zebrafish, MHB constriction requires cell shortening and subsequent laminin-dependent basal constriction of a small ring of cells at the boundary (Gutzman et al., 2008). The cell shape changes involved in MHB constriction morphogenesis require non-muscle myosin II, and recent results show that cell shortening required at the MHB constriction is a consequence of calcium transient regulation of myosin light chain kinase (Gutzman and Sive, 2010; Gutzman et al., 2015; Sahu et al., 2017).

### Maintenance

Once the neural tube is formed and the MHB constriction has been initiated, the genetic program within the MH subsequently transitions to the maintenance phase accompanied by continued reshaping of the brain tissue and ventricular system, as well as production of cerebrospinal fluid that may itself contribute to MHB regulation (Parada et al., 2005; Gato and Desmond, 2009). Computational modeling and experimentation in chick indicate importance of differential myosin-mediated contractility to produce brain ventricle geometry and suggest strategies may differ from compartment to compartment depending on the end fate of the junction, as some are only transient structures (rhombomere boundaries, for example) while others, such as the MHB constriction, persist as structures in the adult brain that must resist increasing fluid pressure from the ventricular system (Filas et al., 2012). One function the MHB constriction may play, thus, is as a point of transition between different anterior and posterior brain ventricle morphogenesis programs converging at the boundary. Such a structure would need to maintain cell adhesion at the boundary until brain ventricle morphology was established on either side to prevent misspecification of the surrounding tissues. The constriction could also mediate the timing of signaling between anterior and posterior brain compartments in the case of signaling molecules secreted in the cerebrospinal fluid. Such phenomena are not without precedent; for example, in mouse embryos it is well known that left/right asymmetry is broken by cilia- directed fluid flow in the node, though it is not known if the signal mediated through the unidirectional fluid flow is mechanical or chemical in nature (Yoshiba and Hamada, 2014). Brain ventricles have been shown to have cilia, and in zebrafish cilia in the developing telencephalon were shown to direct neuronal migration (Kishimoto et al., 2011). Some such mechanism may account for the evolution of the closed primary neurulation strategy seen in zebrafish compared to neural tube infolding seen in other vertebrates.

In the maintenance phase, several sub-regions of the MH emerge that execute their own morphogenetic programs in anticipation of neurogenesis. In the midbrain, the optic tectum is shaped by the formation of a tight sheet of cells called the peripheral midbrain layer (PML) harboring slow-cycling neural progenitor cells that will give rise to columns of neurons organized by alternating protocadherin expression that populate the more anterior tectum in a cortical fashion (Recher et al., 2013; Cooper et al., 2015; Rapaciolii et al., 2016). Posterior to the MHB, the cerebellar rhombic lip and ventricular zones form, from which granule and Purkinje progenitor cells are later derived, respectively, before their neural derivatives organize into the dorsoventral layers and mediolateral compartments that provide the foundation of the cerebellar circuitry (Hashimoto and Hibi, 2012; Millen et al., 2014). The MH tegmentum has almost no overt morphological landmarks apart from a relatively shallow constriction, but its correct patterning and morphogenesis is critical to the proper formation of serotonergic and cholinergic nuclei implicated in important behavioral functions (Parker et al., 2013).

**Figure 2:**
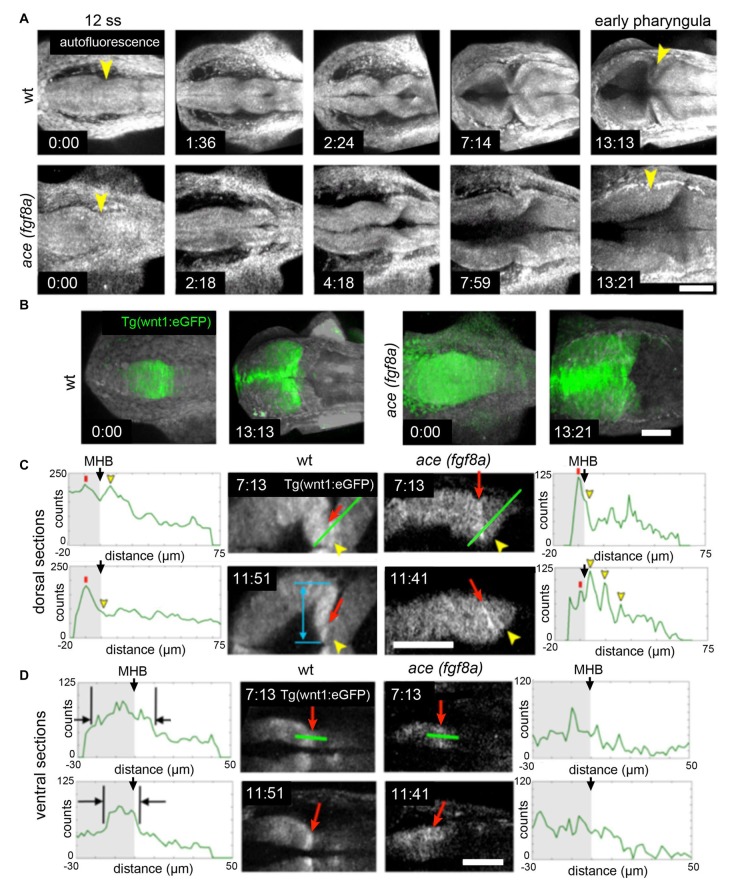
Region dependent *wnt1* reporter response in *ace(fgf8a)* background. **(A)** 3-D reconstructions generated using maximum intensity projection from tissue autofluorescence (with sections from the roof plate removed) reveal the formation of an isthmic constriction (yellow arrowheads) that fails to mature without *fgf8a*. **(B)** *wnt1* lineage is present but improperly polarized in *ace* embryos. During normal development, the *wnt1* lineage increases expression of *wnt1* that can be visualized by increased eGFP reporter signal at the MHB boundary.** (C)** The *wnt1* lineage in the dorsal neuroepithelium normally turns off expression of *wnt1* in the anterior hindbrain as reflected by a decrease in reporter intensity (measured from the profiles marked by green lines over time). Red arrows point to a midbrain cell just anterior to the MHB constriction and yellow arrowheads point to a neighboring cell posterior to the constriction. The blue arrow shows the presumptive peripheral midbrain layer (PML). In *ace(fgf8a)* embryos, the constriction relaxes and neighboring cells in r1 begin re-expressing *wnt1* as shown by increasing reporter intensity, reflecting a cerebellar-to-tectal transformation. Basal constriction of the boundary cells with highest *wnt1* reporter intensity occurs in both cases, however, the presumptive PML fails to form in *ace(fgf8a)* embryos. **(D)** The *wnt1* lineage in the ventral neuroepithelium normally undergoes cell shortening and compresses to a narrow ring of cells anterior to the physical MHB constriction (black markers) while in *ace(fgf8a)* embryos, this reorganization fails to occur and *wnt1* is no longer expressed. Scale bar = 100 μm.

**Figure 3:**
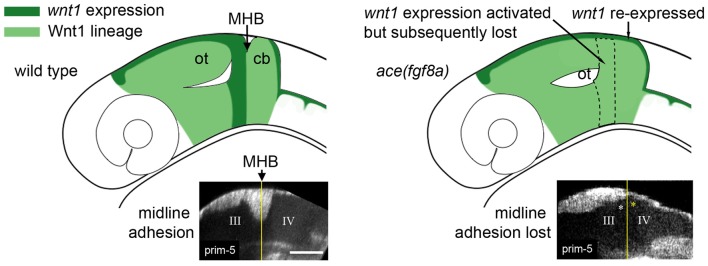
Adhesion failure in *ace(fgf8a)* embryos and regionally dependent *wnt1* lineage response. (Left) Initially broad expression of *wnt1* is normally refined to the dorsal midbrain and anterior midbrain hindbrain domain (MH), helping to maintain *fgf8a* dependent adhesion in the dorsal MH. (Right) *wnt1* expression in the posterior midbrain (except for a dorsal stripe) is lost in *ace(fgf8a)* while *wnt1* expression in a dorsal stripe of r1 is reactivated during a cerebellar-to-tectal transformation. Midline adhesion in the MH is not maintained during brain ventricle morphogenesis.

The correct establishment and maintenance of each of the aforementioned pro-neural sub-regions of the MH requires at minimum a Wnt and Fgf signaling feedback loop to establish the proper unique molecular and mechanical microenvironments (Carletti and Rossi, 2008). In zebrafish *ace(fgf8a)* mutants, the expression of *wnt1* and several other genes in the boundary region including *her5*, *pax2a*, and *eng2/3* are activated but their expression fades as Fgf-dependent feedback fails in early- to mid-somitogenesis (Reifers et al., 1998). Similarly, the combined loss of *wnt1/wnt10b/wnt3a* in zebrafish results in transient expression of *fgf8a*, *pax2a*, and *eng2/3* in the early MHB (Buckles et al., 2004). We have recently found using live multiphoton imaging (Gibbs et al., 2014a) that *ace* mutants form a constriction that fails to mature properly in the maintenance phase (Figure 2), a morphological transient output of a molecular transience (Gibbs et al., 2013). The failure of the constriction to continue morphogenesis in the maintenance phase is due to aberrant cell behaviors in two groups of cells. By imaging a transgenic *wnt1* reporter line (Gibbs et al., 2014b) in the *ace(fgf8a)* background, we identified one group of cells that fails to maintain *wnt1* expression in the posterior midbrain, and to subsequently coordinate the proper morphogenesis of the PML and boundary tegmentum, and another group that fails to suppress *wnt1* expression in the dorsal part of r1 to correctly specify the cerebellar plate (Figures 2, 3). This observation, based on identification of individual cells, supports previous reports of an isthmo/cerebellar-to-tectal transformation in molecular identity of the presumptive cerebellum that occurs with genetic reprogramming during the maintenance phase (Jászai et al., 2003; Gibbs, 2014), though with live imaging we observed that this reprogramming caused by a lack of *fgf8a* does not preclude the previous initiation of the morphogenesis of the MHB during the activation phase. Thus, a mechanism independent of *fgf8a* positions and initializes this physical boundary, while an *fgf8a-*dependent mechanism (either directly or indirectly) maintains its continued morphogenesis.

Wnt and Fgf signaling may also have more direct roles in shaping the MH during neurogenesis. As mentioned previously, during early stages of MHB formation, neurogenesis is actively inhibited by *her5* in zebrafish (Tallafuss and Bally-Cuif, 2003) but subsequently neurons are born as *her5* expression recedes to a narrow ring at the constriction. Wnt1 has recently been proposed to mediate the timing of neurogenesis in the midbrain by driving Fgf8 expression at the boundary and gradually suppressing it away from the boundary by inducing Sprouty expression so that Fgf dependent *her5* also recedes (Dyer et al., 2014). Wnt1 may also function to promote neural stem cell identity in the dorsal midbrain and MHB (Miyake et al., 2012; Lin and Lee, 2016), possibly regulated by Fgf3/8-dependent Fgf22 signaling (Miyake and Itoh, 2013) and may contribute to shaping the MH by regulating the cytoskeleton during axon guidance (Ciani and Salinas, 2005). In the hindbrain, differentiation of unique tegmentum nuclei identities happens in spatiotemporal waves emanating from the upper rhombic lip. Recently, these migrations were shown to be conserved in mice and zebrafish, with discrete Wnt1 populations in the upper rhombic lip sequentially migrating anteriorly toward the MHB and turning ventrally to their final positions in the hindbrain tegmentum (Volkmann et al., 2010). Fgf9/Fgfr2 signaling is important for differentiation of Bergmann glial cells in the cerebellum of mice (Meier et al., 2014), a cell type conserved in the zebrafish cerebellum (although zebrafish do not appear to have an *fgf9*, this function could be attributed to another redundantly functioning Fgf; Bae et al., 2009).

## Morphogenetic Roles for Wnt and Fgf Signaling During Constriction Formation

The Wnt and Fgf signaling pathways are expansive core developmental pathways that play a variety of context-dependent roles. In this section, we further examine the concept that a Wnt/Fgf signaling loop is required for proper MH morphogenesis and discuss potential points of crosstalk between these signaling pathways and cell adhesion and cytoskeletal machinery, based on studies both in the MH and other systems.

### Effect of Modulating Wnt/Fgf Signaling on MH Morphogenesis

Loss of *fgf8a* in the MH in *ace* mutants and subsequent modulation of *wnt1* expression within the *wnt1* lineage (Figures 2, 3) results in a failure of cells near the MHB constriction to carry out certain aspects of normal morphogenesis, including cell shortening, preserving midline adhesion, properly forming the brain ventricles, and organizing the presumptive PML (Gibbs, 2014). Yet, some mechanism (possibly Wnt-dependent) from the positioning or activation phase exists that initiates a transient constriction.

From live imaging, we can deduce that midline adhesion and constriction relaxation are decoupled from each other in the *ace*(*fgf8a)* phenotype, as the time point at which the tracking began in Figure 2C was after adhesion was already lost in r1, yet the orientation of the cells at the boundary changed slowly over time from perpendicular to oblique (opposing the normal orientation) relative to the A/P axis (Gibbs, 2014). We interpret these changes in orientation as reflecting another mechanism that contributes to the *ace(fgf8a)* phenotype independent of adhesion loss. One possibility is a myosin-mediated epithelial relaxation step similar to the mechanism used to create transient constrictions between hindbrain rhombomeres (Gutzman and Sive, 2010), as non-muscle myosins have been shown to be important to MHB constriction formation (Gutzman et al., 2015). Without proper tension in the posterior midbrain epithelium, basal-constricting cells may fail to be mechanically stabilized by surrounding tissues, leading to a loss of proper boundary morphology. We have observed that the *wnt1* expressing cells in the dorsal posterior mesencephalon organize into a simple epithelium at the MHB that coincides with the presumptive PML. Perhaps this organization provides a local mechanical stiffness that allows basal constriction of boundary cells to result in a movement in the anterior vs. the posterior direction. Or, perhaps the basement membrane of that epithelium interacts with the basement membrane of r1 to stabilize the area during basal constriction.

We also tracked similar dynamics in the ventral region of the MH shown in Figure 2D, though signal attenuation at these imaging depths made it difficult to track individual cells (Gibbs, 2014). We observed that the *wnt1* lineage initially spread across the boundary was subsequently compressed into a narrower region as the boundary angle sharpened (black markers). It was not clear if this behavior was a result of basal constriction. In contrast, in *ace(fgf8a)* embryos, the *wnt1* lineage initially spanning the boundary failed to shorten, compress, and organize into a ring of cells, though their orientation did seem to transition from perpendicular to oblique, perhaps as a result of forces resulting from constriction failure in the dorsal neuroepithelium. From this analysis, it would seem there are different mechanical forces exerted on the isthmic constriction along the dorsoventral axis of the tube during somitogenesis, which would potentially lead to different strategies for maintaining the mechanical integrity of the boundary for proper formation of the surrounding tissue.

Previously, increased levels of cell death were observed in dorsal r1 in *ace* mutants during mid-somitogenesis (Reifers et al., 1998) and attributed to apoptosis following an r1-to-mesencephalic identity change. A similar Fgf8^−/−^ phenotype was observed in mice (Chi et al., 2003). While such a phenomenon could potentially explain the loss of adhesion we have seen in dorsal r1, we have not observed any increase in autofluorescence signals that may indicate such events occurring in *ace(fgf8a)* embryos. Instead, we argue that *fgf8a* plays a role in maintaining adhesion during normal development. We also observed in *ace(fgf8a)* embryos that hindbrain opening initiates normally, but spreads anteriorly into the MH, possibly due to the failure to maintain the MHB genetic program without *fgf8a* (Gibbs, 2014). Insufficient Fgf signaling may thus render the dorsal MH competent to a signal emanating from the r1/r2 boundary that triggers hindbrain ventricle opening (Gutzman and Sive, 2010). Interestingly, the limits to which the initially broad *fgf8a* domain narrow (initially spanning r1-r4 and narrowed to discrete domains in r1, ventral r2, and r4; Reifers et al., 1998), mirrors the curious sequence of hindbrain ventricle opening in more posterior rhombomeres. In zebrafish, ventricles open first at the dorsal r1/r2 interface (Gibbs, 2014), followed by small openings at the r3/r4 and r4/5 boundaries (Gutzman and Sive, 2010). The timing of r4 separation reported by Gutzman and Sive (2010) occurs at seemingly the same time *fgf8a* expression is lost in r4 (Reim and Brand, 2002). As forebrain domains of *fgf8a* expression also correspond with areas that shape forebrain ventricle morphology, and a large *fgf8a* domain at the boundary of *otx/gbx* loss of function embryos corresponded with an abnormally long constriction where cells seemed unable to properly delaminate at their apical interface (Su et al., 2014), it is reasonable to hypothesize that *fgf8a* may be a necessary factor to maintain adhesion at brain ventricle boundaries.

**Figure 4:**
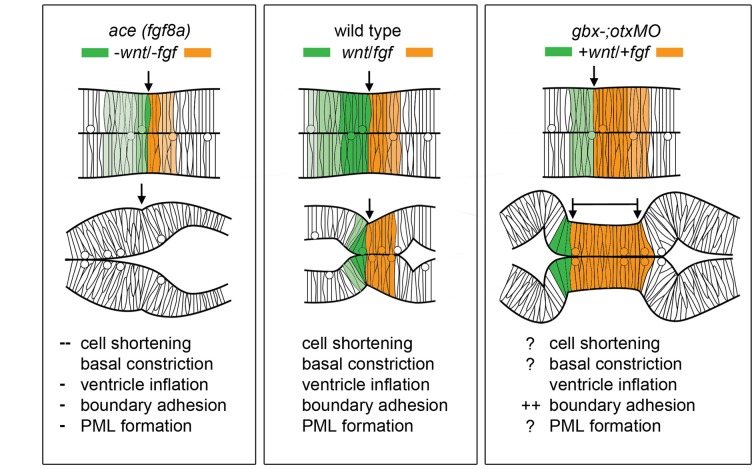
Modulation of Wnt/Fgf signaling and effect on MH morphology. (Left) Summary of *wnt/fgf* expression domains, MH morphology, and morphogenetic cell behaviors in *ace* mutants. (Middle, Right) The same model shown for wild-type embryos and *otx/gbx* loss of function embryos. Figure drawn based on data found in Su et al. (2014). Deficient cell behaviors are indicated with a minus sign (−), while overactive cell behaviors are indicated with a plus sign (+).

Figure 4 summarizes the effects of modulating Wnt/Fgf signaling on morphogenic cell behaviors in the MH during the maintenance phase of formation. Complete knock down of *fgf8a* and transient, low levels of *wnt1* expression in *ace* embryos lead to an opposite adhesion phenotype than that seen when *fgf8a* expression is initiated in an unrestricted manner throughout the MH and subsequently activates late *wnt1* signaling in *otx/gbx* loss of function embryos (Su et al., 2014). The role played by *fgf8a* in maintaining adhesion is likely indirect. Adhesion loss at the midline progresses strikingly in step with the loss of *eng2* and *pax2a* expression patterns in *ace* at the MHB (Reifers et al., 1998) and *eng2*, like *fgf8a*, is not present in r4 when ventricle IV opens there. It would be interesting to examine these patterns in detail to see if *eng2* or *pax2a* are expressed more in cells at the midline and potentially mediate adhesion maintenance. *wnt1*, *eng1.b*, *pax2a*, and *il17rd* overlap with *fgf8a* in that region and would be candidate genes that help to mediate this *fgf8a* dependent separation in the MH. The timing of *wnt1* expression, whether occurring only during the activation phase (as in *ace(fgf8a)*) or during the maintenance phase (as in *otx/gbx* loss of function), may be more important to cell behaviors such as constriction initiation, cell shortening, and PML formation in addition to restricting *fgf8a* anteriorly.

### Wnt/Fgf Crosstalk with Cell Adhesion and Cytoskeletal Machinery

**Figure 5:**
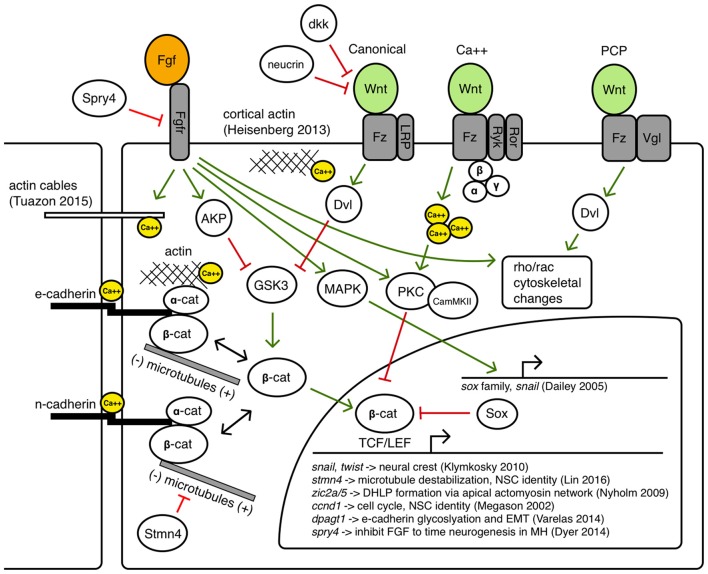
Potential avenues of Wnt/Fgf signaling contributing to MH formation.

Throughout the positioning, activation and maintenance phases of MH formation, it is established that canonical and non-canonical Wnt signaling activity, Fgf signaling activity, as well as precise modulation of cell adhesion, polarity, and motility, is required for correct shaping of the germ layers, the neural primordium, and subsequently the MH. These molecular interactions that may instruct the cell behaviors leading to these morphogenetic changes are summarized in Figure 5. Within the MH, there is likely to be spatiotemporally varying competencies to these interactions, but as they have not been precisely determined in this particular biological system, they are shown together in a single cell as possible avenues for further investigation.

Canonical Wnt signaling has long been associated with the build-up of a pool of cytoplasmic beta-catenin by inhibiting its degradation so beta-catenin can translocate to the nucleus and regulate target genes with TCF/LEF binding sites (Moon et al., 2004). However, both Wnt and Fgf signaling can inhibit GSK3, the primary component of the destruction complex that modulates beta-catenin levels (Dailey et al., 2005). Thus, a careful balance of Wnt/Fgf signaling may affect Wnt target genes during MH development such as *snail* (Yook et al., 2005) and *twist* (Klymkowsky et al., 2010), *zic2a/5* (Nyholm et al., 2009), *cyclinD* (Megason and McMahon, 2002), *stmn4* (Lin and Lee, 2016), and *dpagt1* (Varelas et al., 2014), genes known to affect cell proliferation, dorsolateral hinge-point (DLHP) formation, and adhesion. Classical Fgf signaling may also be important in Bergman glial fate specification in the cerebellum (Meier et al., 2014). Fgf/MAPK signaling can also activate Sox proteins that may inhibit beta-catenin/TCF/LEF interactions, as well as activate Snail, which promotes epithelial-mesenchymal transition (EMT) by inhibiting cadherins (Dailey et al., 2005).

Crosstalk between Wnt signaling and beta-catenin mediated cell adhesion via binding with cadherins has been an area of ongoing research, revealing a large number of context-dependent points of interaction in which Wnt signaling can modulate cell adhesion and vice versa through the shuttling of beta-catenin between a cytoplasmic pool that can become a nuclear effector and a membrane pool that interacts with the cytoskeleton (Heuberger and Birchmeier, 2010). How these interactions may help shape the MHB constriction and other regions in the MH is not known. Zebrafish N-cadherin (*cdh2*) is expressed throughout the nervous system and the *cdh2* mutant *parachute* has significant loss of midline adhesion in the neural tube (Lele et al., 2002). The down-regulation of *cdh2* is accomplished in migrating neural crest cells in a Wnt-dependent manner (Piloto and Schilling, 2010). Zebrafish E-cadherin (*cdh1*) is not expressed in the neural tube until sometime after the 16 somite stage, and is expressed in the presumptive MHB by 24 hpf in a region appearing to overlap with Wnt expression near the midline (Babb et al., 2001). E-cadherin may be uniquely responsible for maintaining cell adhesion to help stabilize cytoskeletal rearrangements at the midline in the MH region or during the formation of the PML. One example linking Fgf signaling with morphogenetic remodeling of the cytoskeleton has been proposed to work through Fgfr-Ras-MAPK signaling in the formation of the lateral line sensory system in zebrafish (Harding and Nechiporuk, 2012). In this study, authors found that Ras-MAPK signaling activated by Fgfr was required for the formation of rosettes by localizing Rho-associated kinase (Rock) to the apical surface to drive its constriction. Fgf signaling has also been shown to have a role in otic vesicle formation, which requires apical constriction mediated by local increases in actin. In the otic vesicle, Fgf signaling activates phospholipase-C (PLC) which triggers non-canonical myosin-II activity (Sai and Ladher, 2008). Classically, myosin-II is understood to ratchet along actin filaments to promote contraction, however, upon phosphorylation by PLC, myosin-II promoted the degradation of basal actin (resulting in enriched apical actin and otic cup invagination). Wnt signaling has also been implicated in cytoskeletal remodeling via the planar-cell-polarity pathway and perhaps also canonical signaling pathways (Lapebie et al., 2011). Once neurogenesis begins in the MH region, it is possible that Wnt/Fgf signaling may modulate neural migration (Knosp et al., 2015). Wnt and Fgf have been implicated in changes in epithelial cell adhesion in neurogenic cranial placodes (Lassiter et al., 2014) and it has been shown that novel Wnt receptors Ryk and Ror can interact with the cytoskeleton to promote axon guidance (Clark et al., 2012).

Balance of intra- and intercellular calcium is another interesting candidate target bridging Wnt/Fgf signaling with cell adhesion and cytoskeletal dynamics (Kim et al., 2011; Tsai et al., 2015). Fgf signaling can promote intracellular calcium release and affect cytoskeletal organization through calcium and calmodulin dependent protein kinases (Schlessinger, 2000), a function that can also be accomplished by Wnt (Babb et al., 2001; Cohen et al., 2008) and may be combinatorial in the formation of the MHB constriction. The findings of Gutzman et al. of calcium transients that appear to drive myosin-dependent cell shortening in the posterior midbrain highlight the potential of such a morphogenetic role for Wnt/Fgf in the MH (Gutzman et al., 2015; Sahu et al., 2017). Additionally, how extrinsic and intrinsic physical forces triggering mechanotransduction pathways may intersect with tissue patterning pathways continues to be an active area of research (Heisenberg and Bellaïche, 2013).

As Figure 4 summarizes, several cell behaviors in the MH region during the maintenance phase appear to depend on Wnt/Fgf signaling, but studies are just beginning to identify which molecular components of the cell adhesion, cytoskeletal, or mechanotransduction machinery are responsible for particular behaviors and have not yet enumerated the direct pathways downstream of Wnt or Fgf that act in this region. Loss of Fgf8a signaling and resulting failure to maintain *wnt1* expression in *ace(fgf8a)* mutants result in the failure of cells to properly shorten at the constriction, aberrant ventricle inflation and morphology in the posterior midbrain, loss of midline adhesion at the constriction, and the failure of the PML to begin to form; yet, basal constriction of the boundary cells does occur. Expanded Wnt/Fgf signaling (with Wnt signaling not becoming active until the maintenance phase) in *gbx1/2* mutants with *otx2* knockdown results in a large constriction due to excessive midline adherence, apparently normal ventricle inflation and unknown effect on cell shortening, basal constriction, and PML formation. Figure 5 depicts potential avenues connecting Wnt/Fgf signaling and possible downstream effectors of these cell behaviors that continue to require further investigation as it remains difficult to study or visualize how multiple signaling inputs are transduced by secondary molecules and downstream effectors to regulate gene transcription, cytoskeletal dynamics, cell adhesion, and subsequent cell behavior *in vivo*.

## New Tools for Addressing An Old Model Organizer

Despite all we know about Wnt and Fgf signaling and MHB development, significant questions, such as how tissue patterning via cell signaling intersects with the generation of morphogenetic force and cell shape change, remain unanswered. One roadblock to significant further progress has been the lack of appropriate enabling technologies to visualize both cell identity and cytoskeletal changes. Classically, morphogenesis and patterning have been studied in a relatively disconnected manner due to technological limits regarding the scale and precision of genetic and embryological manipulations and molecular labels, the spatiotemporal and spectral resolution of imaging systems, difficulty automating sophisticated image processing tasks, and minimal collaboration among developmental biologists, physicists, and engineers. These limitations are all manifestly highlighted by the gaps in understanding regarding the mechanistic patterning and morphogenesis of the MH region. In this section, we briefly discuss recent advances in imaging and image processing technologies we hope will help enable the assembly of a more spatiotemporally comprehensive model of MH patterning and morphogenesis.

### Imaging

The images presented in Figure 2 were acquired with a home-built, ultrashort pulse microscopy (UPM) system configured to render intensity images using two-photon excited fluorescence. In our UPM system, sub-10-femtosecond pulses from a passively mode-locked Ti:Sapphire oscillator are coupled by a dual-axis, galvanometer-driven scanner into an upright microscope. The zebrafish embryos were mounted in agarose wells and submerged for coupling with water immersion objectives. The upright geometry is advantageous for manually aligning the region of interest in the embryo with the optical axis of the microscope. Generated two-photon excited fluorescence is collected in back-reflected geometry by the microscope objective and directed to photon-counting photomultiplier tubes for image rendering. In this configuration, our UPM system is point-scanning wherein images are rendered digitally pixel-by-pixel (Gibbs et al., 2014b).

The experimental configuration of our point-scanning UPM system is similar conceptually to laser scanning confocal microscopy (LSCM) systems, and comparisons between LSCM and two-photon laser scanning fluorescence microscopy (2PM) have been well discussed (Gao et al., 2012). Here, we emphasize two points, those of signal generation and photobleaching. Fluorescence signal used in LSCM is generated following the linear absorption of incident photons; linear absorption may be verified by a linear relationship between incident laser intensity and fluorescence signal. Indeed, “one-photon” fluorescence is a readily observable phenomenon such that signal is generated throughout the excitation beam path within the sample. Thus in LSCM, a pinhole confocal with the object plane is placed in front of the detector to discriminate against out-of-focus signal so that thin optical images may be rendered.

Fluorescence signal used in 2PM is generated following the nonlinear absorption of incident photons, i.e., simultaneous absorption of two photons. Two-photon absorption may be verified by a quadratic relationship between incident laser intensity and fluorescence signal. This nonlinear relationship between incident laser intensity and fluorescence manifests in limiting signal generation to the focus of the beam. Thus in 2PM, fluorescence detection may be optimized for collection because the signal (point) source is assumed to be the focus of the incident beam. This optimization of collection combined with near-infrared excitation leads to a general advantage of 2PM over LSCM to acquire images from greater depths within thick, biological samples (e.g., the MHB throughout the entire DV axis).

Every absorptive event, whether linear or nonlinear, is potentially catastrophic to the emission properties of the fluorophore. Photobleaching is facilitated by the absorbed energy through which photo-induced damage, chemical modification, and environmental factors contribute to fluorophore fading. Since photon energy absorption is essential to the fluorescence process, the potential for photobleaching is unavoidable. In this respect, 2PM is more frugal than LSCM in its use of fluorophores, which can be an important consideration in live cell imaging studies over developmental time periods (e.g., to visualize the development of the MHB lineage restriction boundary).

Recent advances in live cell imaging have led to the development of systems that can comprehensively capture morphogenetic movements and divisions over multiple developmental stages. These recent advances in “in toto” imaging have been aided by the capability to sensitize high resolution microscopy techniques to single nuclei using genetically-encoded fluorescent markers such as fusion of histone and green fluorescent protein, though endogenous signals have been used to image and track every cell in the zebrafish embryo to create a lineage tree through its first 9 cell divisions (Olivier et al., 2010). Localized to nuclei, fluorescent markers of adjacent cells are well separated which is important for their delineation within the crowded environment of the embryo. Lineage tracing in this context then becomes an exercise in tracking progenitors and their progeny, albeit a challenging informatics exercise, especially in tissues with high cell density such as the forming MHB. This challenge has driven advances in imaging technology and computer aided analyses (Peng, 2008) that have revealed, with high spatial and temporal resolution, collective cell migrations (McMahon et al., 2008) and even divisions and movements of every cell within a developing zebrafish embryo over a 24 h period (Keller et al., 2008).

Light sheet microscopy, which has been developed utilizing fluorescence from linear and nonlinear absorption, has recently emerged in the developmental biology community (Keller et al., 2010; Santi, 2011; Weber and Huisken, 2011; Huisken, 2012) and goes by several names including SPIM (selective plane illumination microscopy), mSPIM (m for multidirectional), and DSLM (digital scanned laser light sheet microscopy), denoting differences in configuration and formation of the light sheet (Keller and Stelzer, 2008). The basic principle of SPIM was developed in 1903 by Siedentopf and Zsigmondy (Huisken, 2012), but as with LSCM, the technique did not impact the biological community until much later when in 2004, SPIM was used *in vivo* to image both the relatively transparent medaka embryo and more opaque Drosophila embryo (Huisken et al., 2004). To create a sheet of light, Huisken et al. (2004) used a cylindrical lens, which focuses light along one axis instead of two, as a spherical lens does, creating a sheet of light rather than a line. The sheet is scanned through the sample and the signal is detected by an objective lens placed at a 90° angle that images onto a charged-coupled device (CCD) array. This parallelized excitation and detection renders an entire image onto the CCD camera, greatly increasing acquisition speed especially when compared with point-scanning microscopies.

More recently, light sheets have been created by fast-scanning a laser beam with a long depth of field or confocal parameter along one axis (DSLM), providing significantly higher signal to noise ratios than previous approaches (Keller and Stelzer, 2008). In demonstrating DSLM, Keller and Stelzer (2008) characterized nuclear movements in zebrafish over the first 24 h of development for both wild type and Mzoep mutant embryos. Keller and Stelzer (2008) found that the mechanism of hypoblast formation during epiboly varied by position, with dorsal mesendoderm forming by ingression and ventral mesendoderm by involution. Mzoep mutants failed to internalize cells to form the hypoblast.

In the light sheet configuration, the excitation beam path is in the object plane of detection and, thus, generation of fluorescence signal used to render images is confined to the plane of imaging. Therefore, whether fluorescence is generated by linear or nonlinear absorption, excited fluorophores contribute signal to image rendering before its unavoidable loss to photobleaching. This economical use of fluorophores compares favorably with LSCM that generates signal widely and then discriminates against out of focus fluorescence. In optimizing light sheet microscopy for live cell imaging and minimizing photobleaching in particular, different configurations have been developed to maximize acquisition speed and resolution while homogenizing illumination and minimizing peak intensities, e.g., lattice light sheet microscopy (Chen et al., 2014).

It is now possible to image with high (sub-cellular) resolution and to track the divisions and morphogenetic movements of every cell within a developing embryo over multiple developmental stages. With these technological developments, and with developments in genetic and embryological manipulations to generate genetically encoded molecular labels, constituent specific imaging and characterizing the interactions of multiple constituents in the developing embryo are now possible and may enable multicomponent analysis of complex biological systems. With fluorescence-based microscopy techniques, multiconstituent imaging will require different labels, excitation of those labels, their detection and image segmentation.

Technical hurdles to multiconstituent imaging exist but are rapidly being addressed. One issue is excitation and detection of multiple fluorophores in a single sample. The emission maximum of fluorescence from linear absorption is lower in energy than the absorbed photon and, therefore, spectrally shifted from its excitation wavelength. The detection of fluorescence signal may thus be achieved with the spectral rejection of the excitation laser from the detection path. With multiple fluorophores, it may be possible to tune the excitation to within overlapping absorption spectra of the fluorophores, tune an excitation laser for each fluorophore, or some combination thereof. However, with multiple fluorophores, one or more excitation laser wavelengths may overlap with fluorescence signal. Therefore, in spectrally rejecting the excitation lasers, some signal may be rejected as well. One approach to avoid rejection of signal is to sequentially excite the fluorophores, which also aids in image segmentation, albeit at the cost of image acquisition speed (Valm et al., 2017).

The emission maximum of two-photon excited fluorescence is higher in energy than the absorbed photons. In fact, the excitation wavelength is usually far removed spectrally from the detection window, i.e., near-infrared excitation wavelengths for fluorescence in the visible region. Thus, to excite multiple fluorophores, the central wavelength of the excitation laser pulses may be tuned to within overlapping nonlinear absorption spectra of the fluorophores, one may tune an excitation laser oscillator for each fluorophore (though not cost effective), or some combination thereof. Nevertheless, simultaneous excitation and detection of multiple fluorophores is achievable without any loss of signal from rejecting the excitation laser pulses. One approach is to use an ultrashort laser pulse that has a broad excitation spectrum to nonlinearly excite multiple fluorophores simultaneously (Gibbs et al., 2014a). Segmentation of the images may be achieved through spectral unmixing. This approach has an added advantage of image co-registration because a single laser is used to acquire the multicomponent images. Other schemes have, for example, elegantly used nonlinear optics to generate multicolor images of neural circuit formation in combination with the “brainbow” labeling system (Mahou et al., 2012).

Rapid advances in imaging technologies coupled with new methods for chromosome engineering via CRISPR/Cas9 (for example, see Sander and Joung, 2014) promise to usher in a new period of rapid advances based on these technologies to understand MHB development. For instance, the ability to engineer specific genomic loci to express fluorescent reporters of developmentally important genes will allow a greater understanding of cell lineage behaviors in imaging-friendly organisms. As one example, Ota et al. (2016) used CRISPR/Cas targeting to generate an eGFP expressing allele of zebrafish *pax2a* (Ota et al., 2016). By combining lineage and cytoskeletal reporters in multiconstituent imaging experiments, one could monitor the contributions of specific cell types to MHB morphogenesis. Alternatively, new high resolution labeling and imaging methods could enable the visualization of specific chromosomal loci and enhancers within defined cell lineages to understand the genomic control of cell fates within the MHB. These new tools are rapidly expanding our ability to visualize aspects of MHB development that were previously obscured from view.

### Image Analysis

**Figure 6:**
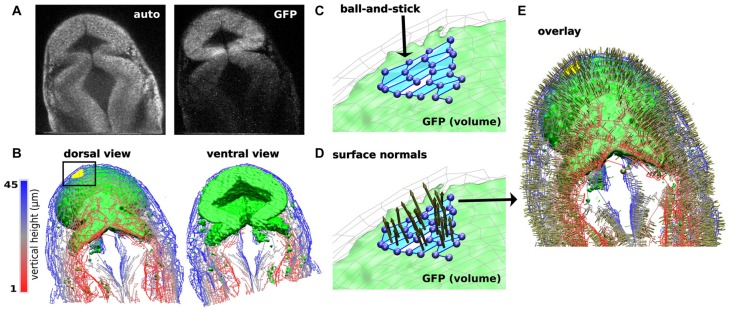
Computer Aided Feature Extraction (CAFE) reconstruction of zebrafish brain embryo images. **(A)** Single slice 2-photon microscope images of zebrafish brain embryo at 20 hpf displaying autofluorescence (left) and GFP (right) channels. GFP channel marks *wnt1* expression. **(B)** Zebrafish embryo structure representation of autofluorescence (wires) and GFP (green volume). Visualizing images 1–45 in the 79-image stack in dorsal (left) and ventral (right) views. Autofluorescence (wires) reconstructed using boundary-based detection function to recognize local pixel gradients and define ball-and-stick model along the edges of the image. GFP (green volume) reconstructed using an area-based detection function to place balls in areas of high pixel intensity. **(C)** Detailed ball-and-stick model of boxed region in **(B)** showing CAFE-defined morphological surface (light blue). **(D)** Local surface normals for surfaces highlighted in **(C)**. **(E)** Dorsal view of overlay of autofluorescence and GFP reconstructions with surface normals.

Another current limitation to characterizing dynamic changes in the developing MHB is the state of image analysis software. The number and breadth of image analysis tools are increasing. Use of these tools ranges from general application programs such as ImageJ and Icy (de Chaumont et al., 2012; Schindelin et al., 2015) to application-specific tools (Peng, 2008; Eliceiri et al., 2012). Development of these tools is driven by the need to rapidly and quantitatively analyze large, multi-modal image datasets. Such data vary in image types, time-series of 2D or 3D images, and multi-channel measurements. There are also many aspects of image analysis, such as visualizing pixel data in more informative ways, making automated measurements when images have distinct features or landmarks, and building structural models of the system being imaged. Despite the plethora of available software, visualizing zebrafish morphogenesis, especially during early stages of development, poses a considerable challenge since distinct landmarks (Mikut et al., 2013) may have not developed yet in these systems. Currently, there is no single software that can process an image stack and render a 3D reconstruction model of the imaging volume. Remarkably, even though the early-stage zebrafish embryo is relatively featureless (landmark-free), humans can easily recognize its shape. The fact that human brains can process the image and construct a mental map of the zebrafish embryo suggests that it is in principle possible to computationally generate a similar 3D model. A 3D model refers to surfaces and anatomical volumes “recognized” or “identified” by a software program and mathematically described so that multidimensional parameterization is possible. This refers to obtaining values for 2- and 3-dimensional features, such as measuring the volume of the brain ventricles and compartments, distribution of gene expression and spatiotemporal evolution, as well as co-registration across different samples. To achieve this goal, we have been developing a program named CAFE (Computer Aided Feature Extraction). It originated from an application mainly to quantify filaments in 2D images (Hwang and Eryilmaz, 2014). A distinct feature of CAFE is its synergy between image analysis and model building. Since pixel data are affected by experimental conditions, CAFE initially uses pixel data to build a coarse-grained, ball-and-stick model of the system being imaged (Figure 6). Working on geometric elements instead of pixel data increases calculation speed and allows for mathematical descriptions of the model. For example, for the ball-and-stick model of a zebrafish brain, another model from a different imaging channel (e.g., fluorescence image stacks of gene expression) can be overlaid, which will be useful for analyzing fluorophore spatial relation (Figures 6A–C). Once the ball-and-stick model is constructed, additional mathematical operations are possible, such as defining surface normals (Figures 6D,E).

To the goal of obtaining a comprehensive spatio-temporal atlas of embryonic development, a tool such as CAFE is indispensable. Ultimately, quantitative description of the development process will be necessary. For example, the evolution of the volume and morphology of different brain compartments at various time points and distribution of gene reporters across the brain and their time evolution are imperative relationships that would vary in pathological development including neurodevelopmental diseases. Further, in order to build a “canonical” model of zebrafish brain development, measurements described above have to be made across images of many different embryos, and averaged at each time point, which requires co-registration (Gibbs et al., 2014b). Building such canonical models is a major goal of structural bioimage informatics.

Analysis between data samples will quantify the variability of morphogenetic changes and provide a defined basis for conclusions in developmental studies. Current image processing tools vary in terms of compatibility with imaging modality, user interface, and extent of automated analysis. Since the zebrafish is an ideal model organism that can be used to classify both gene expression and morphology in vertebrates, there are several software applications developed exclusively for high-content, high-throughput zebrafish imaging data. Applications range from cell tracking to generating a 3D brain atlas of zebrafish larvae, which allow researchers to address questions in developmental biology, neuronal pathways, genetics, etc. (Ronneberger et al., 2012; Mikut et al., 2013; Gibbs et al., 2014b). An advanced zebrafish-specific application is ViBE-Z, a web-based framework that performs colocalization of zebrafish larvae at 48, 72, and 96 hpf (Ronneberger et al., 2012). ViBE-Z requires manual selection of morphological landmarks from a training set of images to perform sequential registration techniques and align test samples to a training set. Using image colocalization, ViBE-Z can segment anatomical regions of the zebrafish larva and visualize gene expression patterns in the zebrafish volume. The restriction on sample time points that can be analyzed, as well as the needed similarity between samples for colocalization, poses a problem when analyzing early-stage (less than 24 hpf) zebrafish embryos with minimal major features or mutant phenotypes. In addition, a major limitation of ViBE-Z, and other zebrafish imaging software, is the lacking potential for quantitative measurements. Obtaining volume-based measurements is challenging since multiple images, a reference coordinate set, and definitive markers at a particular location within a coordinate set are needed. As an example, ViBE-Z appropriately co-registers several image samples and visualizes where, relative to anatomical regions, a gene is expressed in the volume, but it does not measure volume of anatomical regions or distribution of gene expression, nor allow for temporal comparisons.

CAFE services the need in image analysis software tools by building a volumetric model from image stacks based on a Cartesian coordinate system. CAFE uses the reconstructed ball-and-stick model based on the original image’s local pixel intensities to perform image-processing steps, such as increase intensity or rotate the image, as well as obtain measurements of 2D and 3D features. Normals to the surface can be calculated from the surface patches shown in Figure 6C. Figures 6D,E show these surface normals. For volumetric analysis, CAFE can detect the boundary surrounding a user-defined open region and define its general geometry. This parameter is the 3D complement of the midbrain ventricle area parameter derived from 2-D cross sections (Gibbs et al., 2014b). Pixel-based algorithms have been developed and implemented into several image-processing platforms to perform image analysis calculations. CAFE’s ability to produce 2D and 3D ball-and-stick models and use these representations to obtain quantitative calculations will enhance the ability to extract measurements from multiple image samples. Researchers will be able to quantitatively classify spatiotemporal changes in a developing embryo and likewise mark characteristic effects of missing gene reporters, such as in the *ace* phenotype.

## Conclusion

Crucial to understand for its role in brain development and potential contributions to human brain patterning birth defects, the MHB is also a remarkable model for the dissection of signaling control of cell fate and tissue morphogenesis. In this review, we present evidence that the Wnt-Fgf signaling interface is correlated with specific morphogenetic changes that drive MHB morphogenesis. The complexity of MHB patterning and morphogenesis creates an imperative to apply new methodologies and approaches to uncover its underlying molecular nature. The simultaneous generation of correct brain morphology, cell types, and neural circuitry is a daunting challenge but is a very robust and adaptable process. Cell behaviors such as differential adhesion, growth and apoptosis, migration and cytoskeletal remodeling must be precisely coordinated over large regions within the developing neuroepithelium and this achievement is so astounding that it is understandable we have been both fascinated and frustrated with our attempts to understand the process as a whole. Moving forward to a more complete mechanistic understanding connecting the earliest patterning events with eventual brain architecture and cell fates will require enhanced cooperation between disciplines so that the best possible models can be formulated and thoroughly tested. There are still many open questions regarding how correct brain architecture is formed and cell types designated during neural development, though a thorough understanding of these processes is of extreme importance from a basic science perspective and also for advancement of regenerative medicine in neural disease.

## Author Contributions

All authors developed and wrote the manuscript.

## Conflict of Interest Statement

The authors declare that the research was conducted in the absence of any commercial or financial relationships that could be construed as a potential conflict of interest.
